# Public Health Pharmacology: a framework for population-level immunologic monitoring (PLIM) to optimize vaccine performance in Africa

**DOI:** 10.3389/fpubh.2026.1965936

**Published:** 2026-10-02

**Authors:** Ahmed Adebowale Adedeji, Aminah Adeola Adedeji, Einas Mohammed ElHabibi Adam, Taofeek Tope Adegboyega, Issa Muritala Bale, Rasheed Omotayo Adeyemo, Mojeed Akorede Gbadamosi, Hassan Sibomana, Kasim Sakran Abass, Drissa Makan Toure

**Affiliations:** 1 Department of Pharmacology and Toxicology, School of Medicine and Pharmacy, College of Medicine and Health Sciences, University of Rwanda, Butare, Rwanda; 2 Foresight Institute of Research and Translation, Gikondo, Kigali, Rwanda; 3 Department of Epidemiology, Lagos State Ministry of Health, Lagos, Nigeria; 4 Equator University of Science and Technology, Masaka, Uganda; 5 Department of Medical Microbiology and Parasitology, School of Medicine and Pharmacy, College of Medicine and Health Sciences, University of Rwanda, Butare, Rwanda; 6 Department of Primary Care-Palliative Care and Community Health, School of Medicine and Pharmacy, College of Medicine and Health Sciences, University of Rwanda, Butare, Rwanda; 7 Division of Epidemiology and Immunization, Rwanda Ministry of Health, Kigali, Rwanda; 8 Department of Physiology, Biochemistry, and Pharmacology, College of Veterinary Medicine, University of Kirkuk, Kirkuk, Iraq; 9 Division of Epidemiology and Community Health, School of Public Health, University of Minnesota, Minneapolis, MN, United States

**Keywords:** biological cold spots, health equity, population-level immunologic monitoring, public health pharmacology, vaccine performance gap

## Abstract

Despite high administrative immunization coverage across Sub-Saharan Africa, unexpected infectious disease outbreaks pose significant public health challenges. Traditional tracking relies heavily on logistical inputs and reactive surveillance, leaving an unmonitored biological gap in real-world host immunity. This paper presents a novel systems-design conceptual framework for Population-Level Immunologic Monitoring (PLIM) as a core operational tool of Public Health Pharmacology (PHP) to systematically bridge this gap. PLIM operates across three interconnected pillars: (1) biological sentinel surveillance using field-stabilized samples, (2) risk stratification mapping the climatic-biological nexus, and (3) near-real-time policy feedback via digital health infrastructure. To demonstrate operational plausibility, we describe a prospective “Pathfinder Pilot” validation roadmap in Rwanda. This theoretical proof-of-concept model details a logistics blueprint utilizing Dried Blood Spot (DBS) sampling paired with quantitative ELISAs and T-cell Activation Assays (TASA) integrated into existing DHIS2 software to monitor population cohorts against the Population Immunologic Threshold (PIT)—a composite metric linking individual correlates of protection to herd immunity thresholds. Crucially, PLIM complements existing administrative tracking, pharmacovigilance, and epidemiological outbreak investigations. By isolating biological host factors, the framework provides an intermediate biological audit layer. This empowers public health managers to detect waning immunity and map “biological cold spots” before outbreaks occur, advancing immunologic equity across resource-limited settings.

## Background

Vaccination remains one of the most effective tools in combating infectious diseases globally, yet significant challenges persist in translating clinical availability into sustained field effectiveness within Sub-Saharan Africa. Despite widespread immunization initiatives, numerous endemically burdened regions continue to experience outbreaks and waning protection. This persistence suggests potential systemic gaps in vaccine performance that go beyond simple issues of access or coverage.

The discrepancy between trial efficacy and real-world impact is often profound. For instance, the Bacille Calmette–Guérin (BCG) vaccine demonstrates efficacy ranging from 60 to 80% in temperate climates but drops to near zero in equatorial settings, a variation likely driven by environmental mycobacterial interference and parasitic co-infections (1, 2). Similarly, long-term follow-up of the RTS, S/AS01 malaria vaccine reveals that effectiveness is not static; it decreases in high-transmission settings and wanes significantly depending on the demographic and genetic profile of the population (3, 4). Compounding these genetic and environmental vectors are severe nutritional deficiencies, which actively function as biological modifiers of host immunity in resource-limited settings. Chronic micronutrient deficiencies, particularly zinc and vitamin A shortages, mechanically impair lymphoid tissue architecture, disrupt B-cell affinity maturation, and diminish mucosal IgA production, thereby significantly dampening the expected biological yield of routine oral and systemic vaccines (5, 6). The COVID-19 rollout further illustrated this disconnect, where ‘effectiveness’ was often assumed based on foreign trial data rather than verified against local variants and host immune profiles (7, 8). Collectively, these examples highlight a ‘vaccine performance gap’: the divergence between the theoretical biological potency of a vaccine and its actual immunological yield in the ‘last mile’ of African health systems.

To address this complex interplay of host genomic diversity, nutritional deficiencies, and environmental co-exposures, public health systems require an explicit epistemological framework. While PHP interfaces with epidemiology, immunology, clinical pharmacology, and implementation science, it is not merely an intersection; it addresses an unmonitored structural blind spot that none of these parent disciplines examine individually. Epidemiology tracks disease transmission patterns across populations but largely treats the pharmacological agent as a fixed, uniform input. Implementation Science focuses on delivery systems, cold chain logistics, and dose administration (“last-mile access”) under the operational assumption that an intact vial delivered equates to biological protection. Clinical Pharmacology characterizes pharmacokinetic and pharmacodynamic mechanisms, but traditionally restricts its inquiry to controlled, homogenous, and often non-representative clinical trial cohorts. We propose that these systemic biological challenges fall under the domain of Public Health Pharmacology (PHP). Defined in our foundational work as “the discipline that studies the population-level behavior, effectiveness, safety, distribution, and strategic deployment of pharmacological agents within public health systems” (9). PHP uniquely investigates population-level pharmacodynamics. It evaluates how real-world population heterogeneity—including widespread endemic parasitic co-infections, endemic micronutrient malnutrition, regional genetic diversity, and climate-driven physiological stress—modifies the biological mechanism, immunogenic yield, and duration of protection of pharmacological agents deployed at public health scale. Central to operationalizing this discipline is Population-Level Immunologic Monitoring (PLIM).

This discipline of PHP distinguishes itself, as shown in Table 1, from epidemiology (which studies the disease) and implementation science (which studies delivery) by focusing specifically on the *pharmacodynamic interaction* between the agent, in this case, the vaccine, and population-level biology.

**Table 1:** Differentiating the Disciplinary Focus on Vaccine Performance.

Feature	Epidemiology	Implementation science	Public health pharmacology (PHP)
Primary question	Where is the disease?	Did the vaccine reach the child?	Did the vaccine induce the intended biological mechanism? (23)
Primary metric	Incidence and prevalence	Coverage and doses administered	Population Immunologic Threshold (PIT) (10, 19)
View of population	Demographic and geographic clusters	Logistical units and “Last Mile” access	Biological and Pharmacodynamic host environment (24)
Actionable output	Disease containment and outbreak response	Supply chain and logistics optimization	Biological optimization (Boosters/Formulation shifts) (25)

PLIM is structured as a systems-based, theoretical framework aiming to routinely assess vaccine-induced immune responses at scale. It is critical to state that PLIM is designed to complement, not replace, traditional public health pillars, including routine epidemiological disease surveillance, passive pharmacovigilance, conventional vaccine effectiveness studies, and administrative coverage monitoring. While logistics-based tracking captures inputs (doses delivered) and epidemiology captures clinical endpoints (disease incidence), PLIM isolates the intermediate biological variable- population-level immunogenic yield- to provide a mechanistic baseline for real-world vaccine performance. In this paper, we outline the theoretical architecture of the PLIM framework, distinguishing its pharmacodynamic parameters from classic public health metrics, and demonstrating how this theoretical approach is conceptualized to shift African immunization monitoring from an assumption of ‘vaccine access’ to a verified validation of ‘vaccine protection’.

## Methods

### The PLIM framework: a systems approach to vaccine pharmacodynamics

The Population-Level Immunologic Monitoring (PLIM) framework is designed to function as a diagnostic engine of Public Health Pharmacology. Unlike traditional pharmacovigilance, which relies on passive safety reporting or sporadic efficacy studies, PLIM actively and systematically seeks to verify the *biological performance* of vaccines in real-world populations. The framework operates through three interconnected pillars: (1) Sentinel Surveillance, (2) Risk Stratification, and (3) Real-Time Policy Feedback.

### Methodological boundaries: immunogenicity, protection, effectiveness, and impact

To ensure conceptual clarity within the Public Health Pharmacology paradigm, the operational parameters of PLIM would have to be explicitly distinguished from standard epidemiological and clinical trial metrics. In this respect, the following terminologies are clarified.

1. Immunogenicity refers strictly to the intrinsic capacity of a vaccine to elicit a measurable immune response (such as neutralizing antibody titers or antigen-specific T-cell activation) in an individual host environment under controlled settings.
2. Protection represents the physiological state resulting from that immune response, defining whether an individual has achieved a threshold of immunity sufficient to resist clinical infection or severe pathogenesis.
3. *Population immunologic threshold*: While classic vaccinology focuses on an individual Correlate of Protection (CoP)- defined by Plotkin (10) as a specific immune marker statistically associated with protection from clinical disease- Public Health Pharmacology requires a macroscopic, population-level surrogate. We define the Population Immunologic Threshold (PIT) as the critical proportion of a population that must sustain individual immune responses above the validated protective threshold to interrupt community transmission. For pathogens with established humoral correlates (such as measles, rubella, or tetanus toxoid), PIT is mathematically anchored to herd immunity dynamics ( $\mathrm{PIT}=\frac{1-1/R_{0}}{\mathrm{VE}}$ ), where $R_{0}$ is the basic reproduction number and VE is field vaccine effectiveness). For example, measles requires a PIT where ≥93−−95% of individuals maintain neutralizing antibody titers ≥120mIU/mL . However, for complex pathogens lacking a single, binary antibody cut-off (such as malaria or tuberculosis), PIT cannot be treated as an absolute biological guarantee. In such contexts, PLIM utilizes PIT as an empirical, risk-stratified benchmark that combines quantitative antibody concentrations with cellular activation markers (e.g., TASA) to grade relative population vulnerability rather than predict binary sterile protection.
4. Vaccine effectiveness evaluates how well a vaccine prevents specific clinical endpoints within a dynamic, real-world population, factoring in logistics, cold chain compliance, and field execution.
5. Public health impact measures the downstream macro-level societal benefits, such as reductions in crude mortality, changes in transmission dynamics, or the preservation of healthcare capacity.

### Operational distinction from conventional surveillance

PLIM occupies a distinct diagnostic niche across the public health continuum:

- *Routine epidemiological disease surveillance*: Operates downstream and reactively, registering clinical disease incidence, syndromic presentations, and outbreak clusters. It signals a failure after transmission has already propagated.
- *Conventional vaccine effectiveness studies*: Typically utilize observational designs (such as test-negative case–control studies) to calculate real-world protection against symptomatic disease endpoints post-licensure. While essential, VE studies are retrospective and resource-intensive, often evaluating outcomes months or years after programmatic delivery.
- *Serosurveillance/sero-epidemiology*: Predominantly relies on periodic, cross-sectional population surveys measuring cumulative seroprevalence. Its primary utility is charting past natural exposure or macro-level disease penetration.

In contrast, PLIM functions as an active, prospective pharmacodynamic audit. By continuously measuring quantitative immunogenic yield (titers and cellular kinetics) in sentinel cohorts post-vaccination, PLIM isolates the intermediate biological variable between administrative coverage (logistics) and clinical disease (epidemiology). This proactive posture enables health systems to detect waning antibody kinetics and map “biological cold spots” months before clinical breakthrough infections occur.

The PLIM programme focuses its analytical lens strictly on the first two variables; aggregating individual immunogenicity data into population-level trends to map verified physiological protection (Figure 1). It does not replace field-level vaccine effectiveness designs or epidemiological outbreak tracking; rather, it provides the missing biological baseline that explains *why* a vaccine succeeds or fails to achieve its intended public health impact.

**Figure 1:**
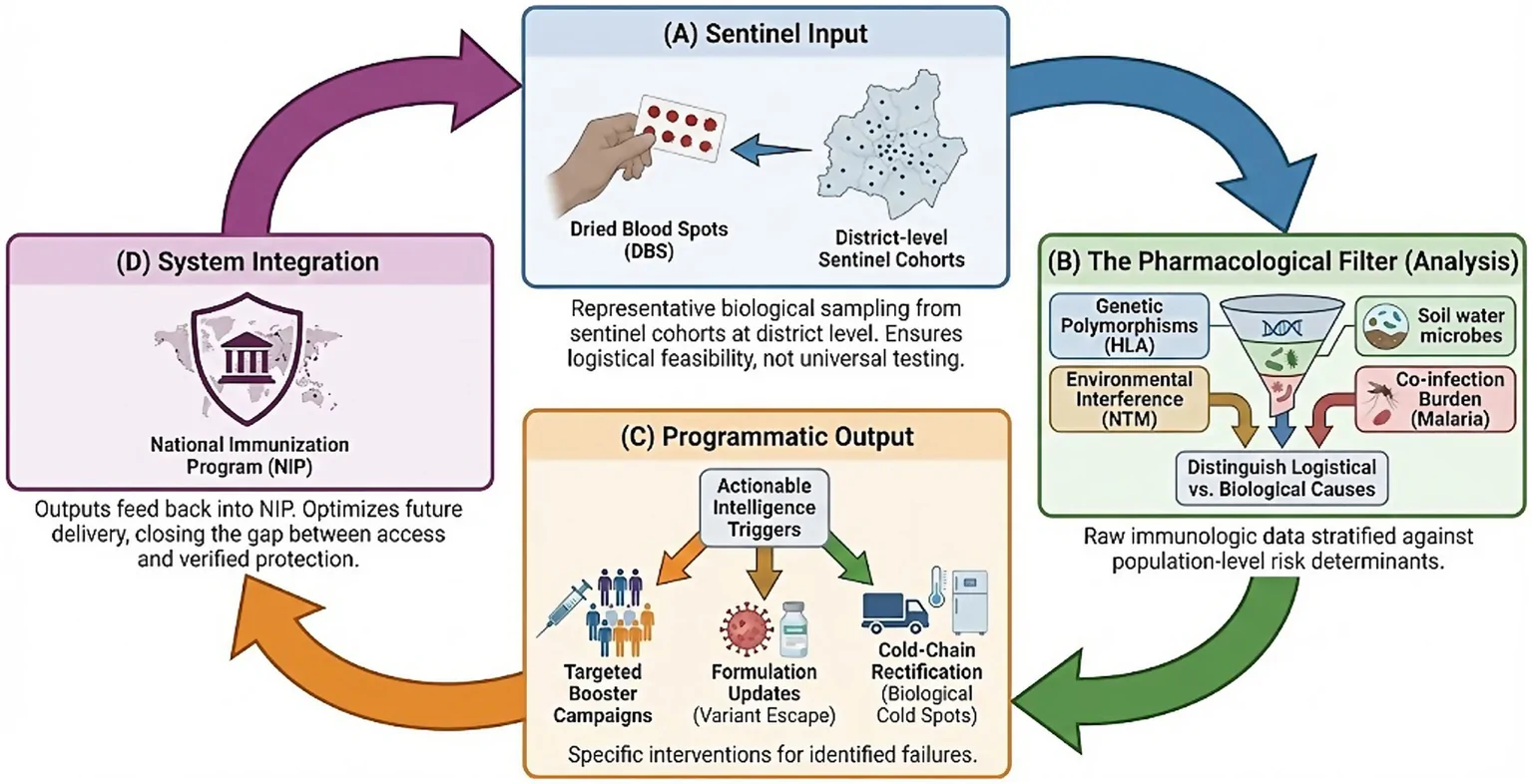
The public health pharmacology cycle of immunologic monitoring (PLIM). A closed-loop system where sentinel immunologic data (Input) is filtered through population risk factors (Analysis) to trigger specific programmatic adjustments (Output). The cycle proceeds in four stages: **(A)** Sentinel Input utilizes feasible sampling methods like Dried Blood Spots (DBS) from representative district cohorts; **(B)** The Pharmacological Filter stratifies this data against biological risks such as genetic polymorphisms (HLA), environmental interference (NTM), and co-infections; **(C)** Programmatic Output generates actionable intelligence (e.g., booster campaigns or formulation updates); and **(D)** System Integration feeds these insights back into the National Immunization Program to optimize future delivery.

#### Pillar 1: sentinel immunogenicity surveillance

To minimize the logistical burden on resource-limited health systems, PLIM moves away from universal testing. Instead, it utilizes a sentinel surveillance model, adapting strategies successfully employed in HIV and malaria monitoring, as shown in Figure 2.

**Figure 2:**
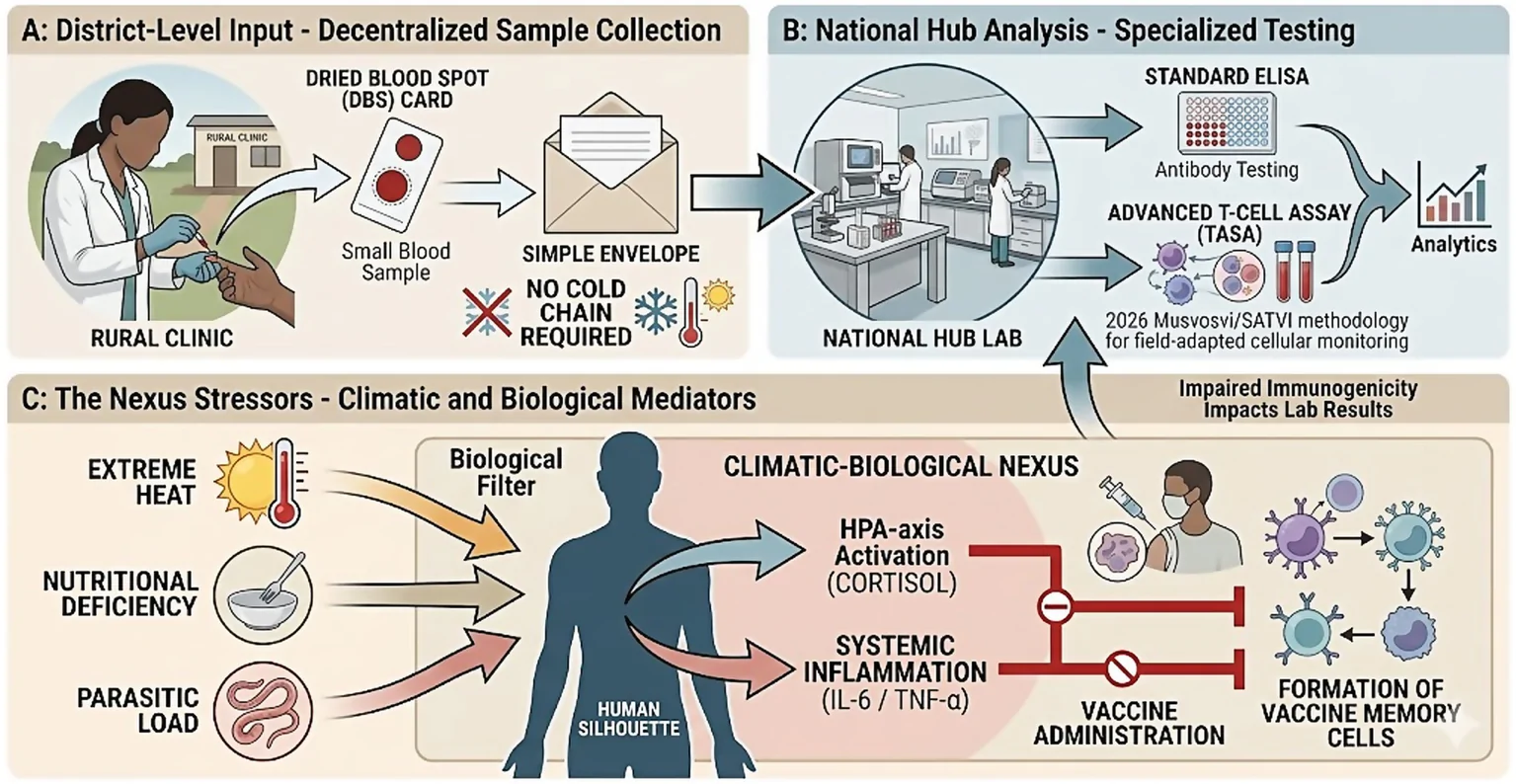
The sentinel immunogenicity surveillance infrastructure and the climatic-biological nexus. Schematic representation of the PLIM Pillar 1 and 2 architecture. **(A)** Decentralized sample collection using dried blood spots (DBS) at the district level, integrated into routine vaccination points. **(B)** The central laboratory analysis phase, highlighting the transition from standard serology to field-adapted T-cell assays (e.g., TASA). (C) The Climatic-Biological Nexus, illustrating how external stressors such as extreme heat, thermal instability, and nutritional shifts, act as biological modifiers that trigger systemic inflammation and compromise vaccine-induced memory cell formation.

##### District level (sample collection and surveillance architecture)

To achieve representative surveillance without overburdening primary health systems, sentinel site selection follows a stratified purposive sampling design across three primary ecological and health-system strata comprising high-density urban settings characterized by high population turnover, variable informal settlement coverage, and elevated transmission velocity; rural endemic regions burdened by high prevalences of parasitic co-infections such as malaria and schistosomiasis alongside documented nutritional vulnerabilities; and agro-climatic thermal stress zones encompassing peripheral districts subject to documented ambient heat extremes and extended last-mile cold-chain transit lines. Within each sentinel facility, representative sampling is linked directly to routine public health touchpoints, focusing primarily on infants presenting for routine Expanded Programme on Immunization (EPI) schedules, including the 9-month Measles-Rubella 1 (MR1) and 15–18 month MR2 milestones, supplemented by pregnant women during routine antenatal care visits. Sampling operates on a dual cadence that combines rapid, rolling cross-sectional bi-annual audits- such as testing 50 consecutive infants per sentinel facility twice yearly- to track macro-level community seroprotection rates against seasonal climatic and nutritional variations, alongside prospective longitudinal cohort tracking utilizing nested cohorts monitored at defined post-vaccination intervals encompassing a Day 0 baseline, 28–35 days post-immunization for peak seroconversion assessment, and 6–9 months post-immunization to determine early antibody decay kinetics. Sample stabilization relies entirely on Dried Blood Spots (DBS), which are validated as a thermostable, cost-effective serosurveillance modality in remote settings that completely circumvents cold-chain transport requirements (11, 12).

##### National reference level (analysis)

Samples are transported to central reference laboratories for analysis. This includes quantitative ELISAs for antibody titers and, where infrastructure permits, field-adapted T-cell assays (such as simplified ELISpot) to measure cellular immunity. Cellular markers are critical, as they often correlate better with long-term protection than antibodies alone, particularly for complex pathogens like malaria and tuberculosis (13). Recent technological advancements now allow for robust, antigen-specific T-cell monitoring using capillary fingerprick samples, effectively bridging the gap between sophisticated laboratory immunology and field-level practicality (14).

##### Integration

These immunologic data points are not siloed but are integrated directly into national health information systems, such as the DHIS2 Tracker. This integration creates a “live map” of population immunity, allowing program managers to visualize protection levels alongside standard coverage rates (15). Recent data synthesis reports from the Rwanda Ministry of Health confirm that the existing digital health ecosystem demonstrates high data consistency and “plausibility” for routine immunization metrics, providing a validated foundation for this immunologic integration (16).

Furthermore, emerging environmental health paradigms suggest that the vaccine performance gap in vulnerable settings may be influenced by the Climatic-Biological Nexus. Environmental shifts, spanning ambient heat extremes to seasonal nutritional fluctuations, represent external stressors that could plausibly modulate host physiology alongside well-documented cold-chain transit vulnerabilities (17, 18). Preliminary physiological evidence indicates that prolonged exposure to high ambient temperatures is associated with systemic inflammatory responses and altered neuroendocrine signaling, raising the biologically plausible hypothesis that chronic thermal stress may influence adaptive immune kinetics, including cellular activation and antibody longevity (17). While definitive causal links between ambient heat exposure and clinical vaccine failure require rigorous empirical validation, current programmatic evaluations remain largely ecological in scope, treating host biological responses as uniform despite widening climatic gradients. Incorporating environmental metrics into PLIM provides a structured platform to empirically investigate these putative biological interactions without overstating causality (17, 18).

#### Pillar 2: risk stratification and response mapping

Once surveillance data is collected, it must be interpreted within its specific biological and environmental context. Public Health Pharmacology recognizes that ‘the population’ is not a uniform biological entity, but rather a dynamic host environment shaped by genetic, nutritional, and climatic stressors. PLIM stratifies surveillance data against these known determinants of vaccine failure (Table 2), enabling health systems to distinguish between programmatic failure (e.g., climate-driven cold chain instability) and biological failure (e.g., host non-responsiveness due to chronic heat stress or co-infection).

**Table 2:** Risk domains and pharmacological indicators of vaccine response variability targeted by PLIM.

Determinant domain	Risk domain	Potential factor	Pharmacological impact on vaccine response	Proposed PLIM indicator/biomarker
I. Biological and host determinants	Genetic	HLA Polymorphisms	Specific alleles (e.g., HLA-B*53) determine the efficiency of antigen presentation, impacting T-cell priming and antibody longevity (26)	Correlation of low titers with specific ethnic or regional genetic clusters.
	Co-Infection	Helminth & Malaria Burden	Chronic parasitic load induces Th2-bias and T-cell “exhaustion,” significantly suppressing vaccine-induced viral/bacterial responses (22).	Stratification of vaccine response data by local parasite burden maps.
	Biological	Host Immunosenescence	Systemic inflammatory signatures (e.g., elevated IL-6, TNF-$\alpha$) induced by stressors impair lymphocyte proliferation and memory T-cell formation (14).	Quantitative T-cell activation assays (e.g., TASA) and inflammatory cytokine profiling.
II. Environmental and external determinants	Environmental	NTM Exposure	Prior sensitization to environmental mycobacteria acts as a “biological block” against live vaccines like BCG, neutralizing immunogenic potential (1).	Baseline interference assays included in TB-endemic zone surveillance.
	Climatic	Extreme Heat Stress	Chronic heat triggers HPA axis activation and elevated cortisol, suppressing B-cell differentiation and antibody affinity maturation (17).	Correlation of PIT (Population Immunologic Threshold) with regional heat-index data.
	Nutritional	Micronutrient Deficiencies	Deficiencies in Zinc, Vitamin A, or protein impair B-cell affinity maturation and mucosal IgA production (6).	Integration of nutritional status proxies (DHS data) with sentinel serosurveillance.
III. Logistical and data-system determinants	Climatic/supply chain	Supply chain instability	Thermal variability increases cold chain “breaks,” leading to protein denaturation or loss of adjuvant potency (18).	Surveillance of vaccine vial monitors (VVM) integrated with real-time immunogenicity results.
	Logistical	Data fragmentation	“Climate-blind” monitoring ignores migration of populations, leading to “zero-dose” pockets and geographic “immunity gaps” (16).	Live-mapping of immunity via DHIS2 Tracker to identify geographic gaps.

#### Pillar 3: real-time policy feedback

The framework culminates in actionable translation, closing the evidence-to-policy loop by converting immunologic surveillance metrics into dynamic programmatic adaptations (Figure 3). Rather than relying on static assumptions, this iterative mechanism systematically accounts for biological and environmental modifiers to preserve population-level protection (8, 18). Because altering national immunization programs incurs significant operational, financial, and regulatory liabilities, PLIM findings do not trigger wholesale policy disruption based on isolated signals. Instead, programmatic responses are governed by an ascending, three-tiered evidentiary hierarchy embedded across three operational policy dimensions:

**Figure 3:**
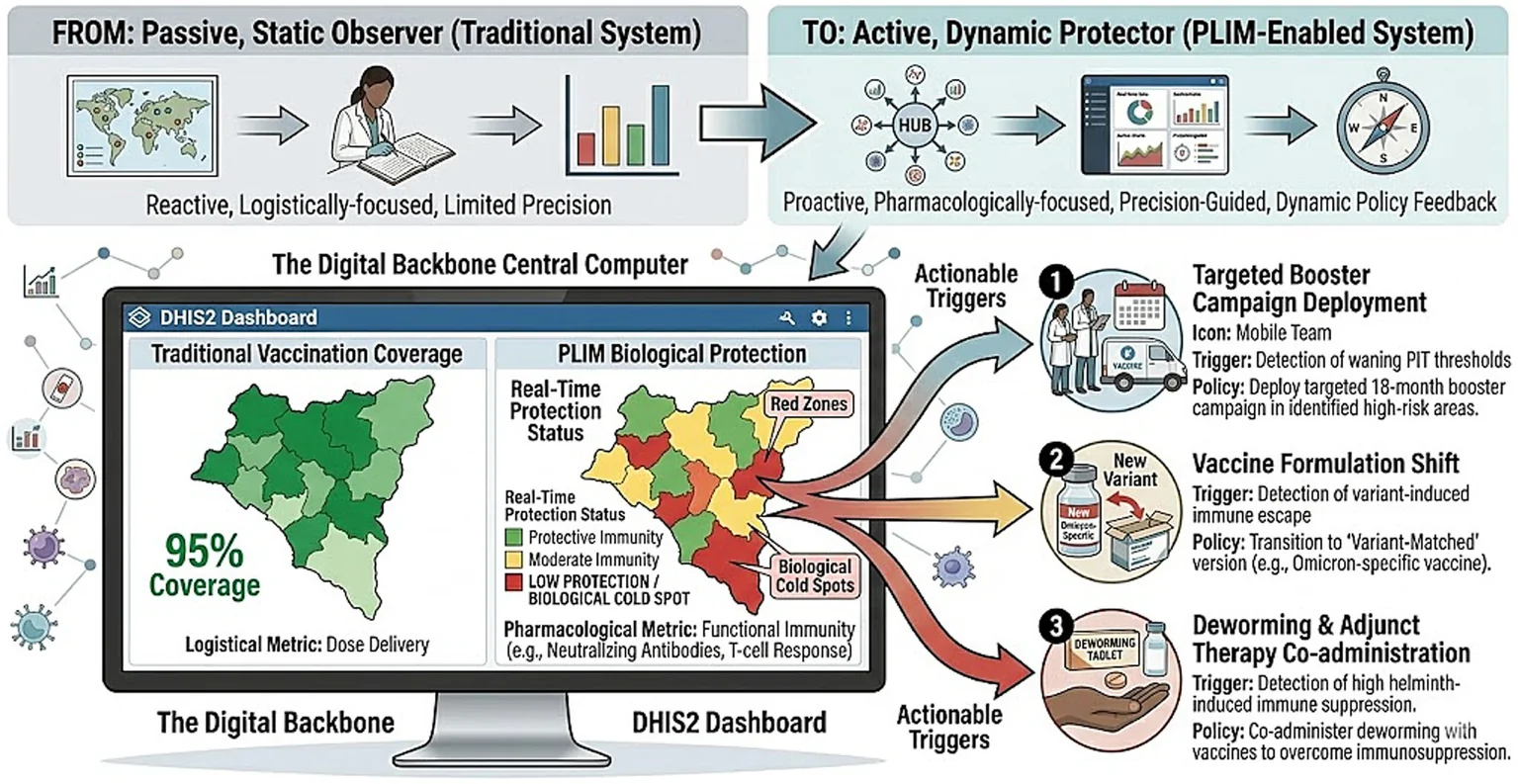
Closing the loop—from static to dynamic policy feedback. Schematic representation of the Public Health Pharmacology (PHP) feedback cycle in action. (1) Real-time integration of longitudinal (post-vaccination) and cross-sectional (sentinel) immunologic data into national health systems like DHIS2. (2) Identification of “Biological Cold Spots” where administrative coverage is high, but population-level protection is low due to variant escape or environmental stressors. (3) Triggered policy actions, including precision immunological scheduling for boosters and evidence-based shifts in vaccine formulation. This iterative loop moves public health toward a precision, climate-resilient framework.

##### Biological equity and operational remediation (tier 1 evidentiary threshold)

PLIM fundamentally redefines health equity by uncovering “Biological Cold Spots”—communities where administrative vaccination coverage appears near-universal, yet population immunity remains compromised by the Climatic-Biological Nexus, such as thermal instability or high parasitic co-infection burdens (8, 18). At this foundational evidentiary tier, localized drops below the Population Immunologic Threshold (PIT) trigger immediate operational remediation rather than national policy shifts. Program managers are alerted to deploy low-risk, targeted investigations, including cold-chain temperature-logger audits, vaccine vial monitor reviews, targeted mop-up clinics, and integrated public health countermeasures such as community deworming or micronutrient supplementation, ensuring health investments are guided by verified biological outcome rather than delivery headcounts alone (8, 18).

##### Precision immunological scheduling (tier 2 evidentiary threshold)

Rather than adhering to rigid, uniform timetables, PLIM provides the empirical rationale for dynamic, data-driven booster timing (16, 19). At an intermediate evidentiary threshold, when longitudinal sentinel surveillance demonstrates consistent, accelerated antibody decay below the PIT across multiple consecutive birth cohorts and distinct geographical clusters, this verified pharmacodynamic signal provides direct documentation for the National Immunization Technical Advisory Group (NITAG). For example, if sentinel monitoring detects that Measles-Rubella (MR) seroprotection falls prematurely below the PIT by 12–15 months in thermally stressed or nutritionally compromised districts, public health authorities can strategically recalibrate routine booster schedules for those specific demographics prior to the conventional 18-month touchpoint (16, 19).

##### Formulation and variant logic (tier 3 evidentiary threshold)

Continuous immunologic monitoring establishes an early-warning mechanism to detect antigen mismatch and variant immune escape (8, 14). Structural modifications to national procurement- such as transitioning to variant-adapted constructs or alternative vaccine formulations- command the highest threshold of evidence. If surveillance demonstrates that a routine vaccine construct fails to elicit adequate neutralizing capacity against circulating local strains, as documented during SARS-CoV-2 lineage divergence, this pharmacodynamic signal initiates a formal verification cascade. To justify procurement shifts, findings require multi-center longitudinal confirmation, cross-validation with regional genomic sequencing data, and policy alignment with Africa CDC and the World Health Organization (7, 8, 14).

## Proposed operational framework

### Operationalizing PLIM in African health systems

Transforming PLIM from a conceptual framework into a functioning public health tool requires deliberate alignment with existing infrastructure. Critics often argue that immunologic monitoring is too costly or technically demanding for resource-limited settings. However, this view overlooks the rapid maturation of African digital health ecosystems and the “leapfrog” potential of decentralized diagnostics. We propose a three-tiered operational strategy: (1) Digital Integration via DHIS2, (2) Laboratory Tiering, and (3) a proposed future Strategic Pilot framework designed to hypothetically evaluate the operational plausibility of Public Health Pharmacology in practice.

*The digital backbone*: Integration with DHIS2 Tracker The primary barrier to PLIM is not the *generation* of data, but its *flow*. Fortunately, the infrastructure to solve this already exists. The District Health Information Software 2 (DHIS2) is currently used for immunization and disease surveillance in over 60 low- and middle-income countries. Indeed, consensus from the 2020 regional workshop in Kigali (20) highlighted that while data availability is improving, the *use* of that data for decision-making remains the critical gap- a gap PLIM is specifically designed to fill. This alignment is timely, as the DHIS2 ecosystem has recently expanded to include dedicated Climate & Health modules, providing the perfect digital architecture to overlay environmental stressors with immunologic outcomes”.

By pivoting the focus from “Doses” to “Protection,” the proposed architecture is designed to build upon the established digital frameworks of the DHIS2 Tracker in Rwanda, for example, which already digitizes individual immunization records and links neonates to specific vaccine batches. We propose the addition of a “Biological Outcome” module to this existing architecture. In this workflow, when a sentinel sample is analyzed, the resulting data, such as a non-reactive Measles IgG status, is entered against the patient’s unique ID. This allows the system to flag vaccine failure at the individual level while aggregating these flags to visualize real-time, district-level performance. This logic extends the recent successes in Mozambique, where interoperable registries mapped “zero-dose” children, to a more granular mapping of “zero-protection” children who fail to seroconvert despite being reached by the system (21).

### Decentralized laboratory architecture: the hub-and-spoke model

To ensure economic feasibility and systemic resilience, PLIM operates on a “Hub-and-Spoke” laboratory model designed to minimize capital costs at the point of care. At the ‘Spoke’ or district level, sentinel health centers are equipped solely for sample stabilization, primarily through the preparation of Dried Blood Spots (DBS). This phase requires no high-tech equipment, significantly reducing the barrier to entry for rural clinics. This decentralized sampling model draws directly upon established, large-scale programmatic precedents across Sub-Saharan Africa, where DBS collection has served as the operational backbone for national early infant HIV diagnosis and extensive malaria seroepidemiology surveys.

By leveraging an infrastructure that primary care nursing staff are already extensively trained in, the proposed PLIM protocol optimizes feasibility and ensures seamless integration into routine primary healthcare vaccination points without requiring complex clinical workflow re-engineering. The technical burden is instead shifted to the ‘Hub’, where analysis is centralized at existing National Public Health Laboratories (NPHLs) or academic centers of excellence.

Centralizing technical expertise and high-cost reagents within a single, quality-controlled environment delivers vital economies of scale without compromising the high analytical sensitivity required for population-level immunologic surveillance. This centralized expertise is particularly vital for the deployment of advanced cellular assays, such as T-cell activation monitoring, which require specialized laboratory protocols (14).

Operationalizing this hub-and-spoke model requires, for laboratory quality assurance, reagent logistics and human resources capacity, a realistic alignment with prevailing laboratory capacities and strict quality governance. At the national hub tier, central reference laboratories—such as those at the University Teaching Hospital of Butare (CHUB) or National Public Health Laboratories—must adhere to ISO 15189 accreditation standards, ensuring that all quantitative ELISA runs incorporate international reference preparations (such as WHO International Standard anti-measles serum) to calibrate optical densities into standardized international units ( mIU/mL ). Internal quality control mandates blinded duplicate testing on 10% of all DBS punches alongside active participation in regional External Quality Assessment (EQA) ring trials. Furthermore, a critical operational distinction is maintained between humoral and cellular assay feasibility. Quantitative ELISA kits for vaccine-preventable IgG antibodies are commercially standardized, stable under standard 2–8 °C refrigeration, widely distributed across African reference hubs, and cost approximately $3–$7 USD per sample. Conversely, advanced cellular assays such as TASA or simplified ELISpot rely on specialized peptide pools, enzymatic reagents, and viable peripheral blood mononuclear cell handling (13, 14). Given reagent supply volatility and technical overhead, cellular assays are not designated for universal or district-level testing; rather, they are restricted to specialized tertiary research centers and performed strictly on a designated 10% sub-cohort for mechanistic validation (14). At the primary healthcare spoke level, nurses require minimal training overhead, as standard operating procedures focus primarily on aseptic capillary collection, precise filter-paper saturation, four-hour ambient drying away from direct sunlight, and humidity-controlled desiccant storage. Because this protocol mirrors established national workflows for infant HIV diagnosis and sickle cell screening, training is readily delivered through concise one-day refresher modules embedded into routine immunization supervisory visits (12).

### Validation roadmap: prototyping public health pharmacology through the ‘pathfinder’ strategy

The translation of the PLIM framework from a theoretical model to a scalable health system intervention necessitates a structured validation strategy. We define a prototypical ‘Pathfinder’ roadmap, proposing Rwanda as a primary sentinel environment for future feasibility assessment due to its advanced digital health maturity and routine immunization coverage exceeding 95%. This strategy is designed not as a standalone study, but as a proof-of-concept for the operational logic of Public Health Pharmacology in endemic settings.

The conceptual design for this proposed validation roadmap leverages the operational integration of the University of Rwanda’s Huye Campus and the University Teaching Hospital of Butare (CHUB) as the analytical reference hub, connected to primary healthcare spoke facilities across Huye District.

*Sample Size Calculation:* The pilot cohort size ( N=500 ) is calculated using Cochran’s formula for population proportion estimation:


$$n=\frac{Z^{2}\cdot p(1-p)}{d^{2}}$$

Assuming an expected seroconversion proportion ( p ) of 0.95 (reflecting Rwanda’s high baseline administrative immunization coverage) (16), a 95% confidence level ( Z=1.96 ), and a precision margin ( d ) of ±2.0% , a minimum sample size of n=456 infants is required. To adopt a conservative design accounting for maximum population variance ( p=0.50 , d=±5.0% , yielding n=384 ) combined with an anticipated 20% loss to follow-up over the 9-month longitudinal observation window, the required enrollment is determined as:


$$N=\frac{384}{1-0.20}=480\approx 500\;\text{infants}$$

*Cohort Follow-Up Schedule:* Enrolled infants receiving their routine first dose of Measles-Rubella (MR1) at 9 months of age are followed longitudinally across three key time points:

- *Visit 1 (Baseline, Month 9):* Capillary blood collected on DBS immediately prior to vaccination to quantify residual maternal antibodies.
- *Visit 2 (Peak Response, Month 10 / Day 28–35 post-vaccination):* DBS sampling to evaluate primary seroconversion and peak antibody titer.
- *Visit 3 (Decay Assessment, Month 18 / Pre-MR2):* DBS sampling immediately prior to the second dose to measure antibody waning kinetics and persistence.

Primary and secondary endpoints:

- *Primary Endpoint:* The proportion of infants achieving seroprotective anti-measles IgG titers ( ≥120mIU/mL ) and anti-rubella IgG titers ( ≥10IU/mL ) at 28 days post-vaccination, and the proportion maintaining seroprotection at 18 months of age.
- *Secondary Endpoints:* Geometric Mean Titers (GMTs); the proportion of complete non-responders; and the correlation between serological decay rates and maternal nutritional status (MUAC) and seasonal ambient temperature indices.

*Statistical analysis plan*: Longitudinal antibody decay kinetics will be modeled using mixed-effects linear regression, treating log-transformed antibody titers as the continuous outcome. Multivariable logistic regression will assess risk factors for failing to maintain the Population Immunologic Threshold (PIT), adjusting for district, nutritional proxies, and ambient heat indices.

Logistically, the sample workflow is structured into three distinct operational phases to ensure system plausibility and cost-efficiency:

1. *Spoke-level capture and stabilization*: At the point of vaccination, a capillary fingerprick sample is collected by routine clinic nursing staff onto a standardized Dried Blood Spot (DBS) card. To minimize operational friction and capital costs at the primary care level, cards are ambiently air-dried for 4 h, sealed in gas-impermeable ziplock bags with desiccant packs, and stored in standard opaque envelopes. This bypasses any need for cold-chain infrastructure during local transport (11).
2. *Consolidated last-mile transit*: Envelopes containing stabilized DBS cards are bundled and transported weekly via existing national specimen courier networks directly to the University of Rwanda/CHUB reference hub.
3. *Hub-level high-throughput analysis*: Upon arrival at the central reference laboratory, a standardized 6-mm punch is obtained from each DBS card for eluate preparation. High-throughput quantitative enzyme-linked immunosorbent assays (ELISAs) are conducted to measure specific anti-measles and anti-rubella IgG titers. Simultaneously, a subset of the cohort (e.g., 10%) will be selected for field-adapted, antigen-specific T-cell Activation Assays (TASA) using simplified ELISpot protocols to establish cellular memory baselines (13, 14).

Rather than functioning as an experimental human trial, this roadmap operates as a structural health system audit. The generated immunologic profiles are mapped against host variables and directly integrated into the DHIS2 biological outcome module (15).

By integrating both this longitudinal verification and ongoing cross-sectional sentinel surveillance, the framework provides a comprehensive “dual-view” of population immunity, capturing both the immediate success of the intervention and the long-term stability of protection. This will allow program managers to visually flag specific communities where a “vaccine performance gap” exists despite perfect administrative coverage tracker metrics.

*Proposed phased implementation architecture*: To address potential economic and policy constraints that may complicate full-scale deployment, the institutionalization of PLIM is conceptualized as a step-wise, progressive implementation strategy. Phase 1 focuses on localized proof-of-concept testing, operating strictly within a single, highly mature digital health district, such as the proposed Pathfinder roadmap tracking Measles-Rubella (MR1) seroconversion across a small sentinel cohort via existing university-linked reference laboratories. Phase 2 introduces risk-targeted scaling, expanding the surveillance network selectively to geographic zones flagged by the Climatic-Biological Nexus as possessing high seasonal malnutrition burdens or extreme heat indices, thereby optimizing resource efficiency. Finally, Phase 3 envisions systemic national integration, executed only after laboratory capacities are consolidated via the hub-and-spoke infrastructure and data pipelines within the national digital health module are validated for decision-oriented practices.

## Discussion

### Toward an era of verified protection

The transition from a global health “access era” to one of “verified protection” requires a fundamental recalibration of our evaluative tools. As immunization systems across Africa evolve from basic delivery models toward precision public health, there is an urgent need to move beyond administrative coverage as the primary surrogate for success.

The PLIM framework addresses this need by operationalizing Public Health Pharmacology (PHP), providing the empirical engine required to move the discipline from theoretical definition to field-level application. Traditional pharmacology often terminates its inquiry at the conclusion of a clinical trial, assuming that “efficacy” under ideal conditions translates linearly to “effectiveness” in the real world. However, as established in the foundational work of this field, Public Health Pharmacology posits that the interaction between a biological agent and a population is inherently dynamic, shaped by a complex interplay of host genomics, environmental stressors, and climatic volatility (9).

In this context, PLIM serves as the “pharmacodynamic monitor” for the last mile. While implementation science focuses on whether the vaccine reached the child, PHP asks the deeper pharmacological question: *Did the vaccine induce the intended biological mechanism within this specific host environment?* This distinction is critical for pathogens like malaria and tuberculosis, where environmental mycobacterial interference and parasitic co-infections significantly alter the pharmacological yield of the intervention (1, 22). By routinely measuring these immune outputs, health systems can finally bridge the gap between laboratory science and field-level implementation.

This shift in focus from “doses” to “protection” carries profound ethical implications for health equity. Current metrics, while logistically useful, can inadvertently mask systemic vulnerabilities. A district may report 95% administrative coverage yet harbor significant “biological cold spots”- pockets of the population where protection may be diminished, potentially associated with climate-driven cold-chain failures or nutritional variances. By relying solely on coverage, we render these unprotected populations invisible. PLIM offers a data-driven pathway to Immunologic Equity, ensuring that high-risk communities are not only reached but effectively protected. Identifying a community where vaccine take is hindered by a high helminth burden, for instance, allows for targeted adjunct interventions—such as integrated deworming and vaccination—rather than the redundant administration of ineffective doses. This strategy ensures that resource allocation is dictated by biological need and verified outcome rather than administrative targets (8).

While PLIM champions the objective auditing of biological outcomes, significant caution is warranted when interpreting humoral titers or cellular markers as direct proof of clinical protection. Biological correlates of protection are inherently probabilistic rather than deterministic. Elevated serum IgG concentrations or detectable antigen-specific T-cell activation indicate an active, functional biological response to an administered antigen, but they do not guarantee absolute protection against clinical infection, especially in the presence of overwhelming viral challenge, mucosal portal entry, or evolving pathogen variants (7, 8, 10). Furthermore, immune protection is often multifaceted; an individual with waning circulating antibodies may maintain robust anamnestic cellular memory sufficient to prevent severe hospitalization while remaining susceptible to mild infection (13, 23). Therefore, PLIM does not claim to deliver absolute biological certainty. Instead, it provides health systems with an intermediate biological probability metric that significantly refines risk estimation beyond administrative headcounts alone.

A critical consideration for the implementation of PLIM across resource-constrained health systems is financial sustainability and cost efficiency. Rather than duplicating parallel structures or introducing unsustainable overheads, PLIM’s economic viability is anchored in two structural efficiencies: capital minimization at the point of care and the preemptive avoidance of reactive outbreak control. By relying on ambiently air-dried Dried Blood Spot (DBS) cards collected at routine immunization touchpoints, the architecture eliminates the prohibitive capital and maintenance costs associated with uninterrupted reverse cold-chain networks required for conventional venous serum transport, reducing field collection and logistics expenditures by up to 70% (11, 12). At the reference hub tier, high-throughput quantitative ELISA platforms utilize standardized commercial assays with marginal reagent costs estimated between $3.00 and $7.00 USD per specimen, while resource-intensive cellular assays are strictly restricted to a 10% research subsample (13, 14). From an economic framework perspective, these modest marginal surveillance costs must be evaluated against the staggering societal and budgetary burdens of reactive outbreak containment. Large-scale reactive campaigns for vaccine-preventable resurgences—such as measles outbreaks—routinely incur emergency operational costs exceeding tens of millions of dollars, compound secondary mortality, and erode public health trust. By delivering prospective pharmacodynamic intelligence that identifies localized waning immunity before community transmission triggers an epidemic, PLIM operates as a high-value, cost-averting surveillance layer that aligns proactive biological quality assurance with sustainable health-system financing (8, 11).

## Conclusion

The ultimate promise of vaccination lies not in the delivery of a vial, but in the generation of sustained immunity. In Sub-Saharan Africa, where diverse immunologic, genetic, and environmental factors converge at the ‘last mile’, this promise is frequently disrupted by a persistent and often invisible vaccine performance gap. We have presented Population-Level Immunologic Monitoring (PLIM) as a strategic architecture to help map this gap. By embedding routine, sentinel immunologic verification into national immunization programs, PLIM transforms Public Health Pharmacology from a theoretical discipline into an operational tool. It empowers health ministries to detect waning immunity trends early, optimize booster strategies dynamically, and ensure that the mandate of ‘health for all’ translates into ‘protection for all’. As the continent moves toward the next generation of vaccines and local manufacturing autonomy, our metrics for success must evolve. While absolute biological certainty remains elusive due to the inherently probabilistic nature of immune protection and context-dependent correlates of immunity, we can no longer afford to ask only, ‘*How many were reached*?’ We must implement the systems required to evaluate, with high epidemiological and biological probability, ‘*How many are protected*?’ PLIM provides the structured, complementary framework to answer that question.
